# Polycomb Binding Precedes Early-Life Stress Responsive DNA Methylation at the *Avp* Enhancer

**DOI:** 10.1371/journal.pone.0090277

**Published:** 2014-03-05

**Authors:** Chris Murgatroyd, Dietmar Spengler

**Affiliations:** 1 Max-Planck Institute of Psychiatry, Molecular Neuroendocrinology, Munich, Germany; 2 Manchester Metropolitan University, Manchester, United Kingdom; Leiden University Medical Centre, The Netherlands

## Abstract

Early-life stress (ELS) in mice causes sustained hypomethylation at the downstream *Avp* enhancer, subsequent overexpression of hypothalamic Avp and increased stress responsivity. The sequence of events leading to *Avp* enhancer methylation is presently unknown. Here, we used an embryonic stem cell-derived model of hypothalamic-like differentiation together with in vivo experiments to show that binding of polycomb complexes (PcG) preceded the emergence of ELS-responsive DNA methylation and correlated with gene silencing. At the same time, PcG occupancy associated with the presence of Tet proteins preventing DNA methylation. Early hypothalamic-like differentiation triggered PcG eviction, DNA-methyltransferase recruitment and enhancer methylation. Concurrently, binding of the Methyl-CpG-binding and repressor protein MeCP2 increased at the enhancer although *Avp* expression during later stages of differentiation and the perinatal period continued to increase. Overall, we provide evidence of a new role of PcG proteins in priming ELS-responsive DNA methylation at the *Avp* enhancer prior to epigenetic programming consistent with the idea that PcG proteins are part of a flexible silencing system during neuronal development.

## Introduction

The hypothalamus harbors dynamic control systems that govern a wide range of homeostatic processes including energy balance, reproduction, and the response to stress. Structurally, the hypothalamus is a complex neuroendocrine tissue composed of different neuronal cell types that express classical neurotransmitters and specific neuropeptides that act within the hypothalamus and other brain regions to coordinate central and systemic functions.

Developmental studies in mice, chick and zebrafish have evidenced that sonic hedgehog (Shh) signaling plays an important role in the induction and early patterning of the hypothalamus (*for review see* [1]). Neuronal precursors giving rise to the paraventricular (PVN) and supraoptic (SON) nuclei of the hypothalamus first appear in the mouse between embryonic days 10 (E10) and 12 (E12). A portion of these neurons develop into the PVN while others migrate laterally to form the SON between E13.5 and E14.5 [2]. Neurons from these nuclei project their axons to the posterior pituitary where they secrete the two neurohypophyseal hormones arginine vasopressin (AVP) and oxytocin (OXT) in the general circulation.

AVP plays a critical role in the regulation of fluid homeostasis, blood pressure and the response to stress; the latter through its stimulatory effects on the hypothalamic pituitary-adrenal (HPA) axis (*for review see* [3], [4]).

Experimental studies in rodents have demonstrated that early-life stress and sustained stress in adulthood leads to enhanced expression of hypothalamic Avp and an increase in the number of Crh-containing neurons that co-express Avp (*for review see* [5]). These findings suggest that Avp contributes at a given stage of life and in a stressor-specific manner to the regulation of the HPA axis. Consistent with this view, we have previously demonstrated that early-life stress led to heightened stress responsivity in juvenile and adult mice through epigenetic marking at the downstream enhancer region of the *Avp* gene [6].

Molecular mechanisms that mediate epigenetic regulation of gene expression comprise DNA methylation, chromatin (re-) modeling and histone modifications (*for review see* [7]). DNA methylation refers to the covalent bond of a methyl group at the cytosine base of CpG dinucleotides catalyzed by DNA-methyltransferases (DNMTs) and generally serves to repress gene activity on the long term. While *Avp* methylation at the downstream enhancer did not differ between the PVN and SON, early-life stress dependent neuronal activity selectively prevented binding of the epigenetic reader Mecp2 (methyl-CpG binding protein 2) in the PVN and resulted in increased *Avp* expression. Following on, reduced Mecp2 occupancy at the *Avp* enhancer facilitated DNA demethylation and thus gave rise to an enduring memory trace of the initial stress event [8], [9]. In view of these findings, we sought to investigate in the present study the basic mechanisms that lay the ground to *Avp* enhancer methylation and thus enable lasting regulation by early-life stress.

The repressing activities of methylcytosine (5 mC) are mediated by methyl-CpG-binding domain proteins (MBDs), which recruit chromatin modifiers such as histone deacetylases (HDACs) or methyltransferases (HMTs). Another group of proteins with an important role in gene repression and development are the polycomb (PcG) proteins. They assemble as multiprotein complexes containing the core components EZH2 (enhancer of zeste homolog 2), SUZ12 (suppressor of zeste 12 homolog), and EED (embryonic ectoderm development). Moreover, due to their association with HDACs and DNA-methyltransferases (DNMT) these complexes can confer transcriptional repression and stable silencing of target genes.

To investigate the molecular events that contribute to ELS-responsive DNA methylation at the *Avp* enhancer, we used here an embryonic stem cell (ESC) derived model of hypothalamic-like differentiation. Whereas neuropeptide expression patterns were distinct from mice hypothalamus, *Avp* enhancer methylation faithfully evolved upon differentiation. Moreover, consistent with the hypothesis that polycomb proteins (PCG) are part of a flexible silencing system during neuronal development, we evidence a new role in preceding experience-dependent gene expression.

## Materials and Methods

### Animals

C57BL/6, Mecp2 wildtype or Mecp2 null male (-/y) mice [10] were housed under 12 h daily illumination (lights on at 06:00) with food and water ad libitum. All procedures on animals were approved by the Regierung von Oberbayern and were in accord with European Union Directive 2010/63/EU.

### Cell culture

Rx-GFP K/I EB5 cells, a subline of the mouse embryonic stem cell line EB5 (Riken cell bank depository; http://www.brc.riken.jp/inf/en/index.shtml), were maintained in Dulbecco's modified Eagle medium (DMEM), 1% fetal bovine serum (FBS), 10% KnockOut Serum Replacement (KSR), 0.1 mM non-essential amino acids (NEAA), 1 mM sodium pyruvate (all from Invitrogen Gibco), 0.1 mM mercaptoethanol and 2000 U/ml mouse leukemia inhibitory factor (Lif; Millipore). For hypothalamic-like differentiation, dissociated cells were grown as floating aggregates in growth-factor free medium containing DMEM/F12, 1X chemically defined lipid concentrate (Invitrogen), 5 mg/ml bovine serum albumin (BSA, Invitrogen) and 450 µM alpha-monothioglycerol (MP biochemicals) on low cell-adhesion culture wells for 7 days. Cyclopamine (10 µM, Biozol) or purmorphamine (5 mg/ml, Alexis) were added on the 4th day. Cells were cultured in DMEM/F12, 7 g/litre glucose and 10% KSR for a further 3 days. Hereafter, half the medium was replaced with DMEM/F12, 7 g/litre glucose, N2, B27 and 10 ng/ml recombinant rat ciliary neurotrophic factor (CNTF, Biozol) and grown for a further 15 days during which one half of the volume of culture was changed every other day, following the protocol of [11]. Cells were also treated with 0.5 µM or 5 µM 3-Deazaneplanocin A (DZNep) (Cayman).

### RNA extractions and RT-PCR

Total RNA and DNA were simultaneously extracted [12] and reverse transcription (RT) reactions were done on 1 μg of total cell culture-extracted RNA or 100 ng of tissue-derived RNA with SuperscriptII (Invitrogen) and poly-dT primer. Quantitative PCR (qPCR) was performed on a LightCycler (Roche) using LightCycler Fast Start DNA Master SYBR Green (Roche). Primer sequences for qPCR reactions are listed in Table S1 in File S1. For all RT-PCR experiments the expression of target genes was normalized to the geometric mean of expression of three housekeeping genes [hypoxanthine phosphoribosyltransferase (*Hprt*), glyceraldehyde-3-phosphate dehydrogenase (*Gapdh*), and ATP synthase-coupling factor 6 (*Atp5j*)] to minimize variations due to different experimental conditions.

### Bisulfite sequencing

Genomic DNA (200–400 ng) isolated from PVN tissue punches, hypothalami or cultured cells was digested with EcoRI, sodium bisulfite converted (Qiagen DNA methylation kit), PCR amplified and cloned into pGEM-T vector; at least 20 independent recombinant clones per reaction and animal were analyzed on an ABI Prism 3700 capillary sequencer. Bisulfite primers used for qPCR analysis are listed in Table S2 in File S1.

### ChIP experiments

Chromatin from cultured cells, mouse PVN punches (individual pools formed from groups of 3) or whole hypothalami dissected from fresh brains were cross linked, disrupted by sonification (Diagenode Bioruptor™), and purified with the Magna ChIP G kit (Millipore) as previously described [13]. Antibodies used were anti-Suz12, anti-histone H3K27me3 (trimethyl-histone H3, Lys-27), anti-histone H3K9me2 (dimethyl-histone H3, Lys-9) (each from Diagenode; CS-029-100, pAb-069-050, pAb-060-050), anti-H4Ac (panacetyl-histone H4) (Upstate; 06-866), anti-Rpol (activated RNA polymerase II) (Abcam; ab5131), anti-Dnmt1, anti-Dnmt3a (both from Acris; AM20060PU-N, SM7028P), anti-Dnmt3b, anti-Hdac1, anti-Hdac2 (each from Abcam; ab13604, ab46985, ab51832), anti-5mC and anti-5hmC (both from Active Motif; 61255, 39770). ChIP primers used for qPCR analysis are listed in Table S3 in File S1.

## Results

### Differentiation of neuronal progenitors into *Avp*-expressing neurons

In order to study basic mechanisms contributing to ELS-responsive DNA methylation at the *Avp* enhancer, we chose a cellular model of early hypothalamic-like neuronal differentiation. Sonic hedgehog (Shh) plays a crucial role for the coordination of diencephalic tissue growth with anteroposterior (AP) and dorsoventral (DV) patterning and the development of the PVN and supraoptic nucleus (SON) from the lateral hypothalamus [14]. In accord with these findings, hypothalamic-like precursors derived from the embryonic stem cell line Rx-GFP K/I EB5 (hereafter referred to as EB5) can be differentiated into rostral (Shh-treated) or dorsal (Shh-depleted) hypothalamic-like progenitors that subsequently give rise to ventral-medial and PVN/SON-like neurons [11].

**Figure 1:**
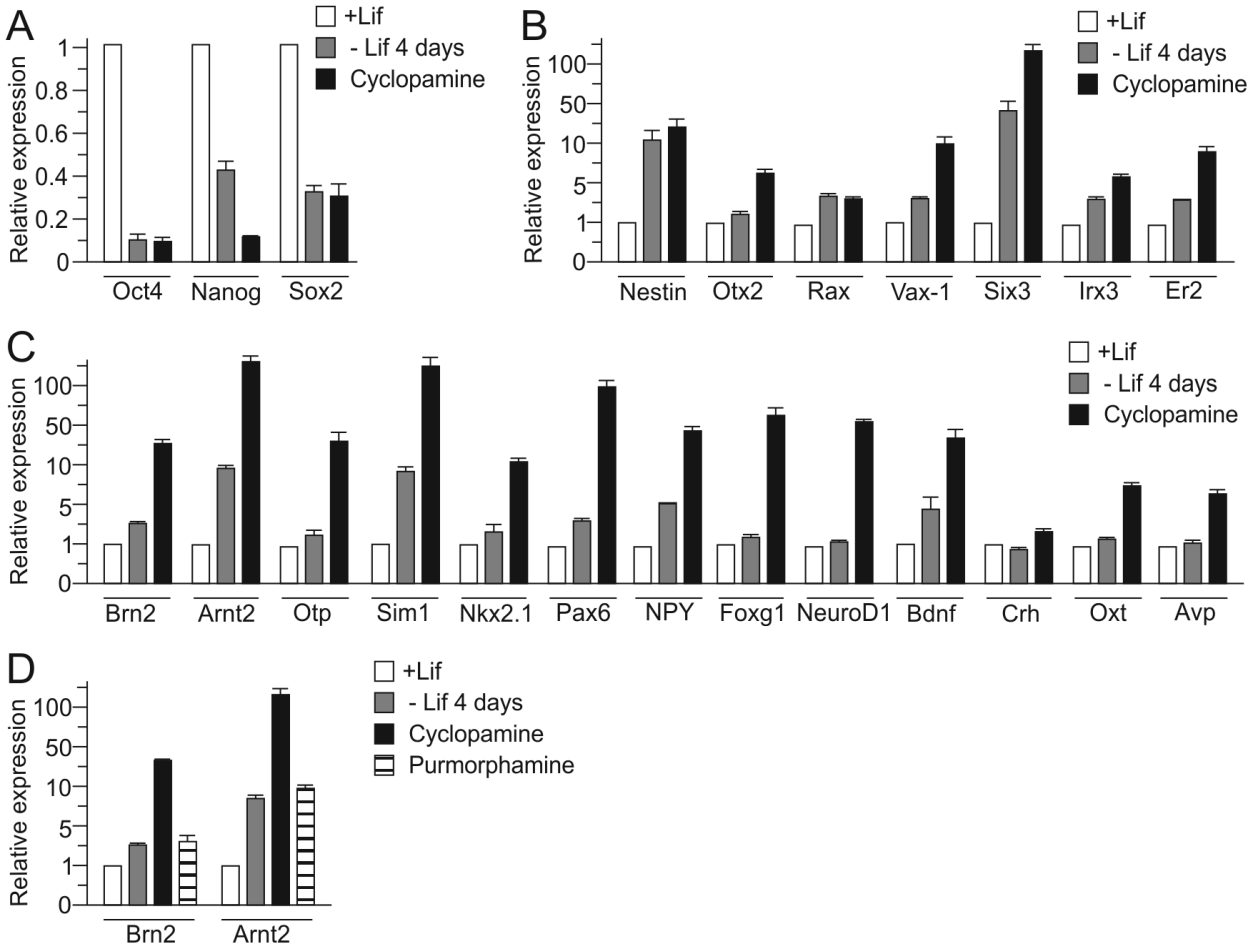
Hypothalamic-like differentiation of the embryonic stem cell line EB5. A, Pluripotency factors rapidly decreased in differentiating EB5 cells over 4 days following withdrawal of leukemia inhibitory factor (-Lif) as evidenced by qRT-PCR analysis. B, Markers for neuroectoderm and rostral hypothalamic development conversely increased during EB5 cell differentiation. C, qRT-PCR analysis of markers for dorsal-ventral hypothalamic patterning and PVN neurons demonstrated strong increases following 25 days of differentiation. D, PVN markers (*Brn2* and *Arnt2*) further increased following treatment with the sonic hedgehog (Shh) antagonist cyclopamine as evidenced by qRT-PCR. Application of the Shh agonist purmorphamine blocked this increase. Bars represent standard deviations from the mean (sem) from at least 3 independent experiments.

Cultivation of EB5 cells in suspension under serum free conditions and in the absence of insulin or other exogenous growth factors resulted in the rapid down-regulation of pluripotency factors (Figure 1A) followed by the up-regulation of hypothalamic proneuronal factors (Figure 1B). Moreover, treatment with the Shh antagonist cyclopamine promoted increased expression of genes specific for the dorsal hypothalamus and the PVN, including the neurohypophyseal peptides Avp and Oxytocin (Oxt) (Figure 1C). The importance of Shh inhibition was corroborated by application of a Shh agonist (purmorphamine) that inhibited the expression of markers characteristic for the PVN such as *Brn2* and *Arnt2* (Figure 1D).

Together, these experiments suggested that hypothalamic-like differentiation of EB5 cells can serve as a suitable model to investigate molecular mechanisms underlying *Avp* enhancer methylation.

### DNA methylation of the *Avp* enhancer during hypothalamic-like differentiation

**Figure 2:**
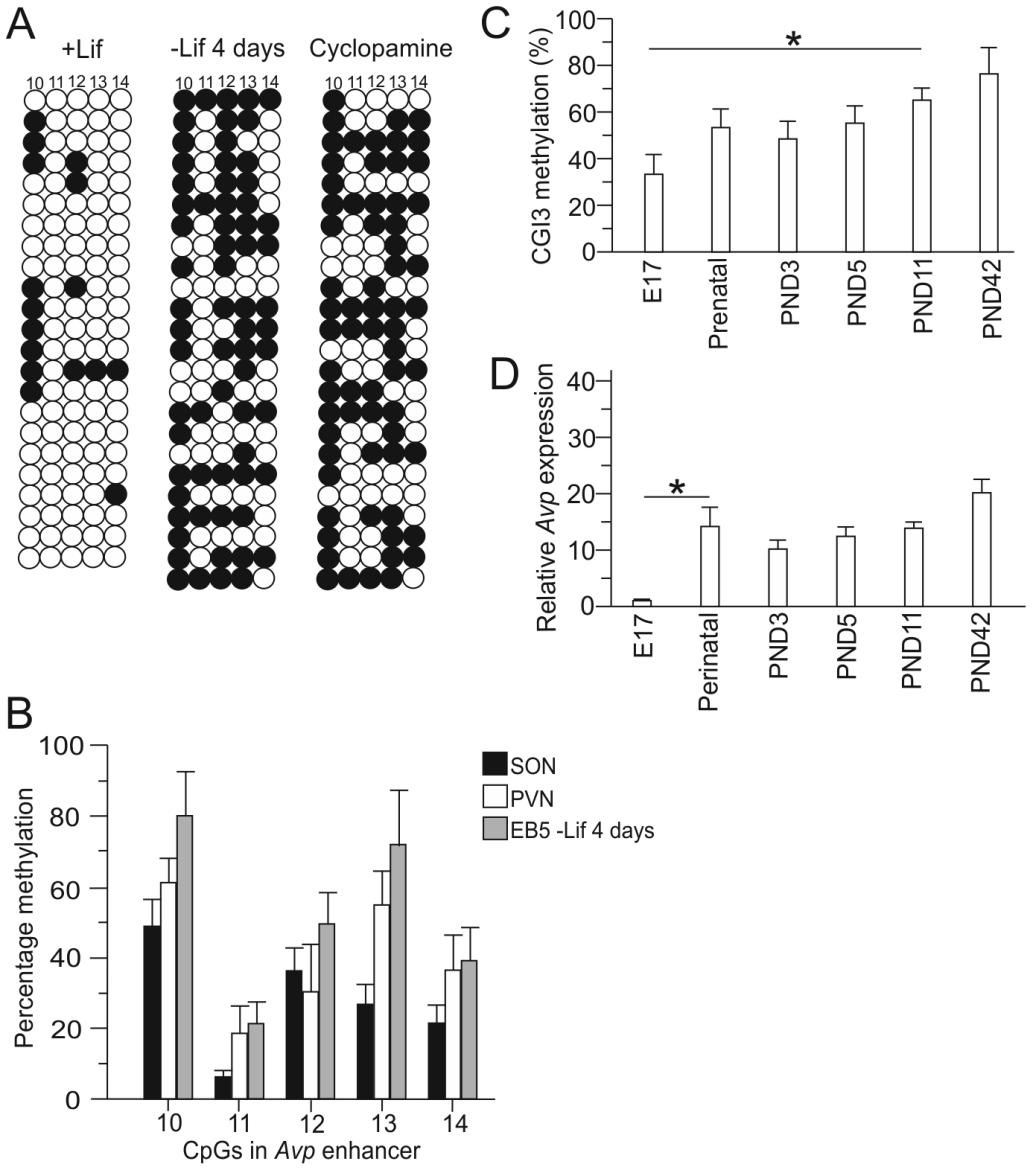
Hypothalamic-like differentiation triggered ELS-responsive DNA methylation at the *Avp* enhancer. A, ELS-responsive DNA methylation rapidly evolved following 4 days of Lif withdrawal and was maintained during subsequent cyclopamine treatment as evidenced by bisulfite sequencing of 25 clones. Shaded hemispheres represent percentage of methylation at each ELS-responsive CpG residue. Results are representative of three independent experiments. B, Comparison of ELS-responsive DNA methylation at the Avp enhancer in the SON, PVN and following hypothalamic-like differentiation of EB5 cells. C, Overall CpG methylation at CpG island 3 (CGI3) comprising the ELS-responsive *Avp* enhancer in the developing mouse hypothalamus. Postnatal days (PND) are indicated. D, Avp mRNA expression as evidenced by qRT-PCR analysis in the developing mouse dorsal hypothalamus. Bars represent standard deviations from the mean (sem), from 4–5 independent experiments.

Having established a cellular model for hypothalamic-like development and *Avp* expression, we sought to characterize the events that precede the appearance of ELS-responsive DNA methylation at the *Avp* enhancer. Analysis of DNA methylation at the ELS-responsive CpG residues revealed a strong increase in methylation following differentiation through withdrawal of Lif, (Figure 2A) which was maintained following cyclopamine treatment. Importantly, *Avp* enhancer methylation of Lif-withdrawn EB5 cells closely matched the methylation profiles from mouse PVN and SON (Figure 2B), [6]. Moreover, the promoter and gene body of the *Avp* gene remained unmethylated during all phases of hypothalamic-like differentiation of EB5 cells consistent with the in vivo findings in mice (data not shown). Therefore, DNA methylation at the *Avp* locus seemed to be conserved between different Avp-expressing brain nuclei (i.e. PVN versus SON) and an in vitro model of hypothalamic-like differentiation. However, Avp expression was undetectable during early hypothalamic-like differentiation and depended on further treatment with the Shh-antagonist cyclopamine which favors the formation of PVN/SON-like neurons (Figure 1C) [11].

As controls, for proper DNA methylation during hypothalamic-like differentiation, we compared the results from the *Avp* gene to those from the pluripotency marker *Nanog* and two classes of repeat elements. The *Nanog* promoter showed a strong increase in DNA methylation following 4 days of Lif withdrawal (Figure S1 in File S1) that correlated with a corresponding reduction in gene expression (Figure 1A) [15]. Similarly, the *LINE1* repeat [16] element strongly gained in methylation, as previously reported. In contrast, methylation at the *IAP* element [17] remained unchanged irrespective of either treatment (Figure S1 in File S1).

Together, these results indicate that methylation of the *Avp* enhancer emerged prior to and irrespective of gene expression during early hypothalamic-like differentiation. At opposite, the *Nanog* gene and the *LINE1* elements were barely methylated in their on-states, while increased DNA methylation associated with their off-states. Such gene silencing serves on the one side in case of *Nanog* to shut-off a pluripotency gene during differentiation and on the other side to restrain the potential for self-replication of *LINE1* elements which undergo uniform methylation in differentiated somatic tissues.

Consistent with the results from above, DNA methylation appeared at the *Avp* enhancer at embryonic day seventeen (E17) to increase a further 20% until birth and a further 20% thereafter until 6 weeks of age (Figure 2C). Hereafter, *Avp* methylation gradually declined with age [18].

*Avp* expression was undetectable at E17, increased peri- and postnatally and increased further in adulthood with the greatest significant change for Avp occurring between E17 to the perinatal time point (Figure 2D). Thus, ELS-responsive DNA methylation precedes *Avp* expression in vivo in agreement with the results from early hypothalamic-like differentiation of EB5 cells. Moreover, increases in *Avp* expression concurred with higher CpG methylation at the enhancer. At odds with these findings, DNA methylation is, however, generally viewed to silence gene transcription.

### Histone marking of *Avp* during hypothalamic-like differentiation

**Figure 3:**
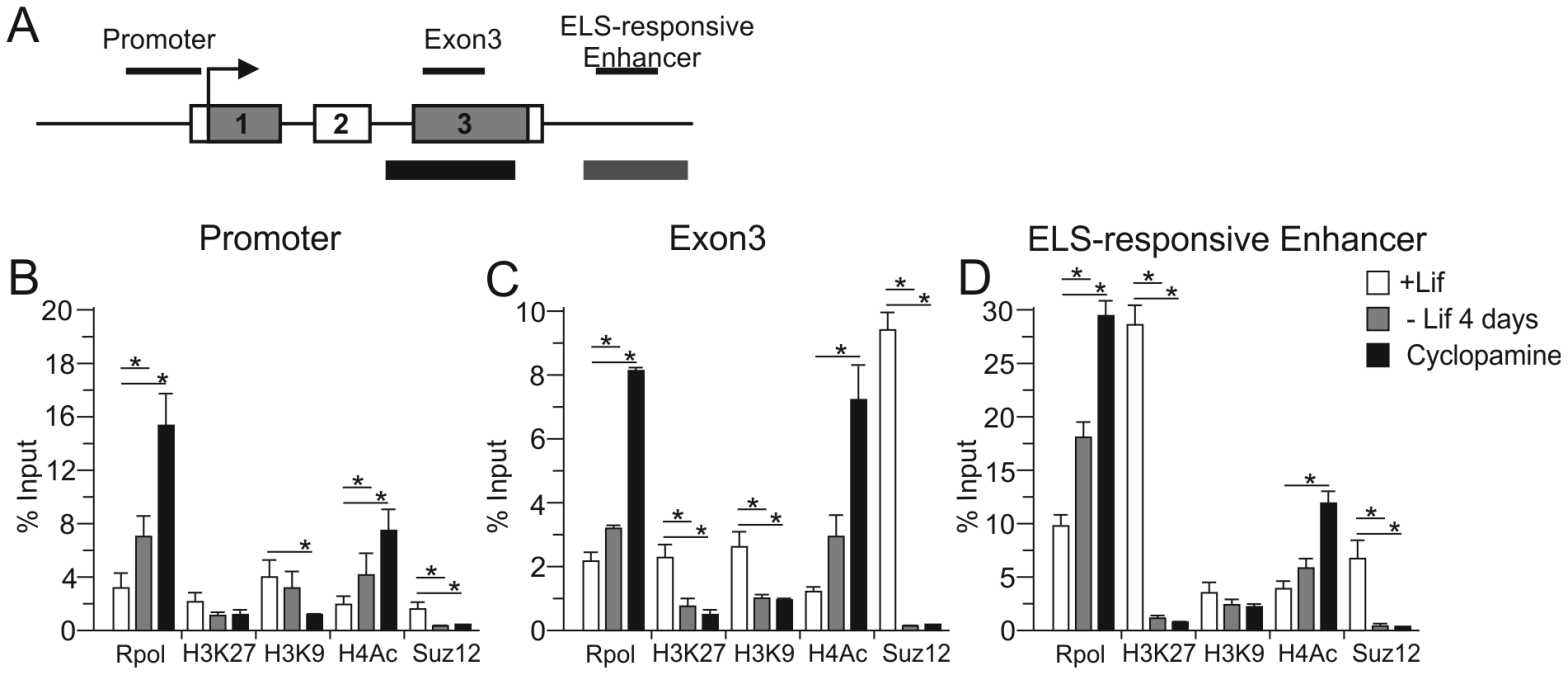
Hypothalamic-like differentiation displaced repressive histone marks and Suz12 occupancy at the ELS-responsive *Avp* enhancer. A, Schematic diagram of the *Avp* locus. Exons are shown as boxes and numbered with coding parts shadowed. Amplified DNA segments depicted above. Arrow marks translational start codon. ChIP analysis was done for the promoter, gene body and downstream enhancer regions. The strong-CpG island at the gene body and the intermediate-CpG island harboring the ELS-responsive enhancer are shown beneath by black and grey bars, respectively. B–D, Chromatin samples from undifferentiated (+Lif), Lif-withdrawn (-Lif) and further cyclopamine treated EB5 cells were immunoprecipitated with antibodies against activated RNA polymerase II, Suz12, H3K27me3 (trimethylation-histone H3, Lys27), H3K9me2 (dimethylation-histone H3, Lys 9), and H4Ac (pan-acetylated-histone H4). Recovered DNA was analyzed by qPCR for the presence of the *Avp* promoter (B), gene body (C) or enhancer (D). Bars represent standard deviations from the mean (sem),*, *P*<0.05; from 4 independent experiments.

The repression of *Avp* expression together with low levels of methylation at the enhancer in undifferentiated EB5 cells suggested the presence of a mechanism that prevents gene transcription before the emergence of ELS-responsive DNA methylation. We therefore investigated the role of another regulatory mechanism, namely post-translational histone modifications. These marks form complex signatures controlling open and closed chromatin configurations corresponding with activation and repression of gene transcription, respectively. We used chromatin immunoprecipitation (ChIP) experiments to track changes in histone marks at the *Avp* locus during hypothalamic-like differentiation of EB5 cells (Figure 3A). At the *Avp* promoter low levels of the repressive histone marks H3K27me3 (trimethyl-histone H3 Lys-27) and H3K9me2 (dimethyl-histone H3 Lys-9) decreased concomitantly to an increase in the active mark H4Ac (panacetyl-histone H4), (Figure 3B). A similar picture emerged for the gene body with more pronounced changes in either mark when compared to the promoter region (Figure 3C). Furthermore, high levels of H3K27me3 at the enhancer region strongly declined concurrently to a robust increase in H4Ac (Figure 3D). The transition from repressive to active chromatin marks correlated with the recruitment of activated RNA polymerase II (Rpol) across the *Avp* gene indicative of the formation of an open chromatin structure permissive to transcription.

Together, these findings suggested the presence of a repressive chromatin domain at the *Avp* enhancer that seemed to prevent precocious expression prior to DNA methylation. Therefore, we next sought to identify the mechanisms directing the establishment of repressive histone marks at the *Avp* enhancer.

The histone mark H3K27me3 is a hallmark of the polycomb repressive complex 2 (PRC2), a class of polycomb-group proteins (PcG), that play a major role throughout development by dynamically switching on and off gene expression programs through interactions with the epigenetic machinery [19]. The methyltransferase Suz12 is part of the PRC2 complex and represents the catalytic subunit that maintains histone methylation patterns throughout DNA replication due to its ability to bind unmethylated DNA and mediate gene silencing through methylation of H3K27 [20].

Therefore, we asked whether the presence of H3K27me3 relates to Suz12 occupancy at the *Avp* locus. Suz12 binding was barely detectable at the *Avp* promoter (Figure 3B) whereas high levels of binding occurred at both the gene body and enhancer (Figure 3C, 3D) and strongly decreased in either case following differentiation.

The binding of Suz12 to wingless (*Wnt1*), a beta-catenin dependent developmental regulator and to the RNA polymerase II promoter (*RNAPII*), a housekeeping gene, served as positive and negative controls, respectively, in these experiments (Figure S2 in File S1) [21].

### Tet proteins prevent ELS-responsive DNA methylation

**Figure 4:**
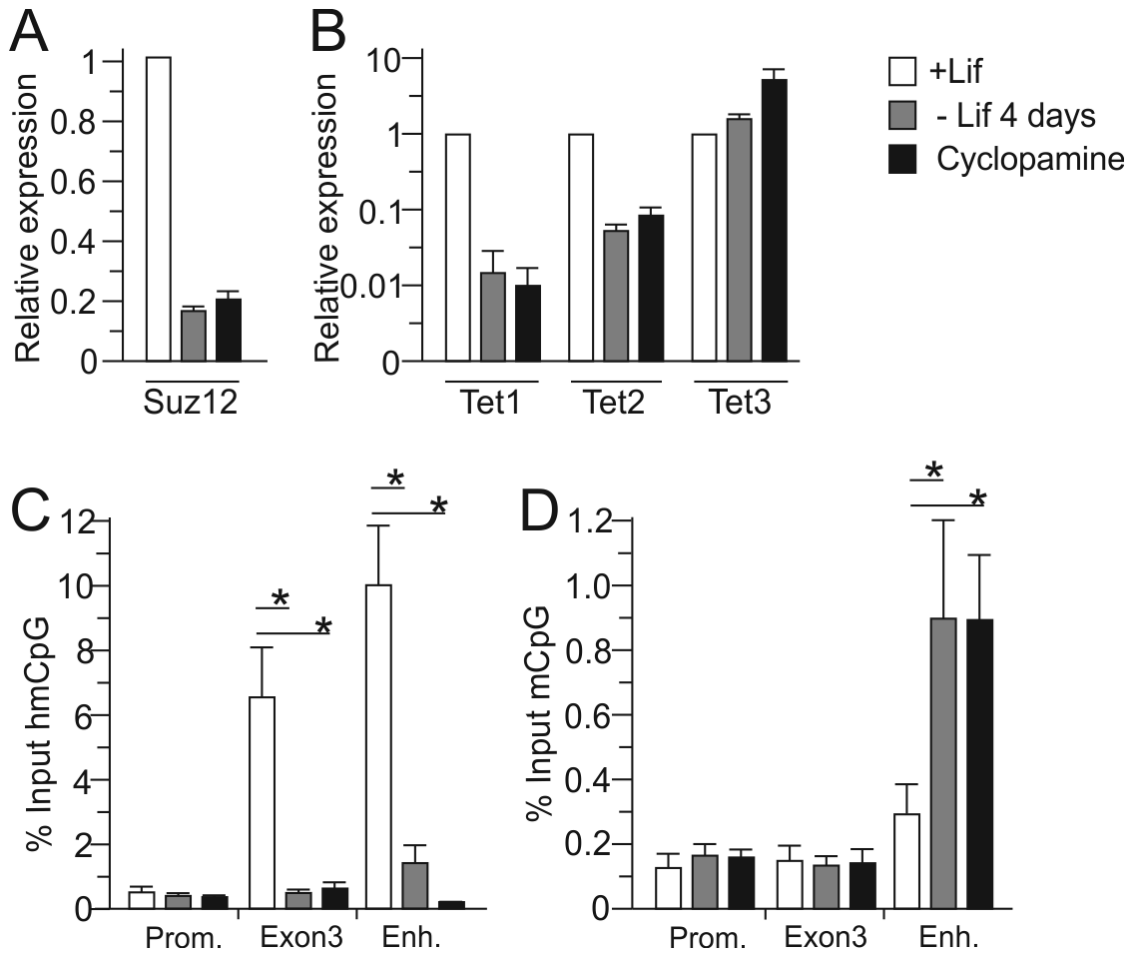
Expression of *Suz12* and *Tet* genes correlated with the presence of methylcytosine (5mC) and hydroxymethylcytosine (5hmC) at the *Avp* locus during hypothalamic-like differentiation. A, *Suz12* gene expression rapidly decreased following 4 days of Lif withdrawal. B, Similarly, *Tet1* and *Tet2*, but not *Tet3*, gene expression rapidly declined following 4 days of Lif withdrawal. C, MeDIP showed high hydroxymethylation (5hmC) at the *Avp* gene body and downstream enhancer which strongly decreased upon Lif-withdrawal. Application of cyclopamine evoked a further decline at the enhancer region. D, Conversely, methylation (5mC) at the *Avp* enhancer, but not at the gene body, increased as evidenced by MeDIP. Bars represent standard deviations from the mean (sem), *, *P*<0.05; from3 independent experiments performed in duplicate.

Having established the mechanism of *Avp* repression in undifferentiated neuronal progenitors, we next sought to understand how the ELS-sensitive enhancer region is primed for subsequent DNA methylation. During hypothalamic-like differentiation, expression of *Suz12* decreased by 5-fold (Figure 4A) whilst its 10- and 2-fold decreased binding at the *Avp* enhancer and *Wnt1* promoter, respectively (Figure 3B and Figure S2 in File S1, respectively) was suggesting an active mechanism by which Suz12 is excluded from the former. With respect to the *Avp* enhancer, one likely mechanism is the onset of DNA methylation at this region, which is thought to interfere with Suz12 binding [20].

The Tet (*ten eleven translocation*) protein family members – comprising Tet1, Tet2 and Tet3 – share the capacity to convert 5-methylcytosine (5mC) into 5-hydroxymethylcytosine (5hmC) to initiate a process of active DNA demethylation. In addition to the conserved catalytic subunit, Tet1 and Tet3 also have a DNA-binding domain previously described as a CpG-binding motif [22]. Hereby, Tet1, being mainly expressed in ESCs, contributes to gene silencing by facilitating recruitment of PcG proteins to CpG-rich gene promoters.

We found that during hypothalamic-like differentiation of EB5 cells *Tet1* sharply decreased, as was the case for *Tet2*, while *Tet3* increased slightly in expression (Figure 4B). We then used immunoprecipitation of methylated DNA (MeDIP) to assess changes in methylation using antibodies against 5mC and 5hmC. Consistent with a role in DNA demethylation, 5hmC, the product of Tet proteins, decreased rapidly at both the *Avp* gene body and enhancer during differentiation followed by an increase in methylation only at the enhancer (Figure 4C, 4D).

Together, these data suggest that PCR2 occupancy at the *Avp* gene body and enhancer depends on the enzymatic activities of Tet proteins preventing precocious DNA methylation. In turn, neuronal differentiation appears to trigger the eviction of PcG proteins concomitant with the encroachment of DNA methylation at the enhancer region.

### DNA-methyltransferase and Mecp2 binding at the ELS-responsive *Avp* enhancer

DNA methylation is catalyzed by a family of DNMTs, classified either as de novo methyltransferases that recognize previously unmethylated CpG sequences, or maintenance methyltransferases that copy preexisting methylation marks onto new DNA strands during replication.

**Figure 5:**
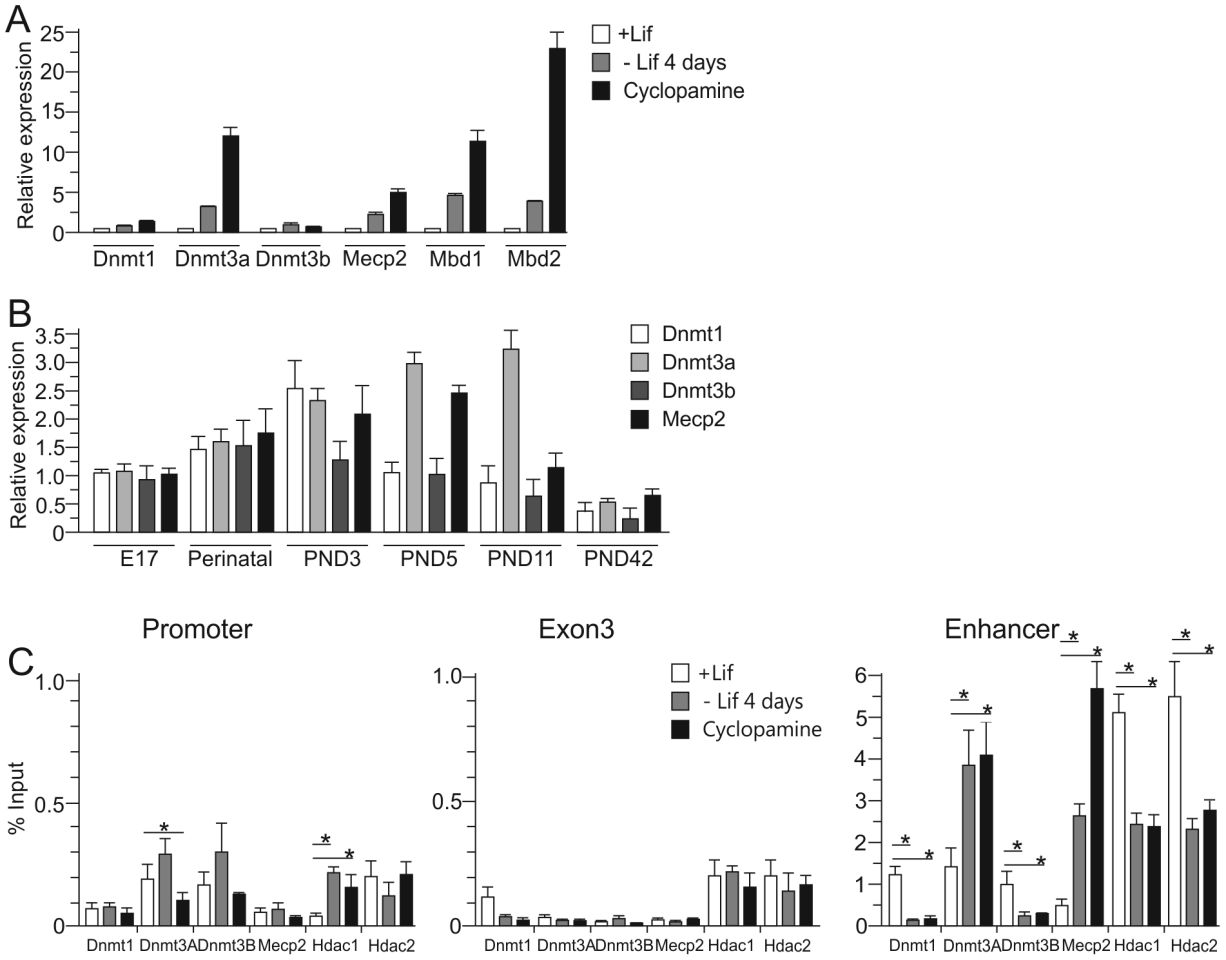
Dnmt3a and Mecp2 associated at the *Avp* enhancer during differentiation. A, *Dnmts, Mecp2*, *Mbd1* and *Mbd2* mRNA expression during hypothalamic-like differentiation of EB5 cells as evidenced by qRT-PCR analysis. B, Expression of *Dnmts* and *Mecp2* RNAs in the mouse hypothalamus. C, Binding of Dnmts, Mecp2 and Hdacs at the *Avp* locus during hypothalamic-like differentiation. Chromatin samples from undifferentiated, Lif-withdrawn and further cyclopamine treated EB5 cells were immunoprecipitated with antibodies against Dnmt1, Dnmt3a, Dnmt3b, Mecp2, Hdac1 and Hdac2. Recovered DNA was analyzed by qPCR for the presence of the *Avp* promoter, gene body or downstream enhancer.

Consistent with the scenario from above that differentiation associates with the accumulation of DNA methylation at the *Avp* enhancer the expression of the *de novo* methyltransferase*Dnmt3a*, increased during EB5 cell differentiation and in the postnatal hypothalamus (Figure 5A, 5B). In contrast, the expression of the maintenance methyltransferase *Dnmt1* was largely unaffected under both conditions. Importantly, Dnmt3a occupancy increased selectively at the *Avp* enhancer while binding at either the promoter or gene body remained unaltered (Figure 5C). This finding demonstrated that increases in Dnmt3a expression were not sufficient to promote *per se* DNA methylation but seemed to require selective recruitment to the enhancer region.

In accord with this idea Dnmt3a occupancy at the *Avp* enhancer did not change following cyclopamine treatment (Figure 5C) which led to a further increase in *Dnmt3a*expression (Figure 5A). Together, these results indicated that Dnmt3a recruitment at the *Avp* enhancer was “actively” controlled and took place during the formation of hypothalamic-like progenitors.

The family of methyl-CpG-binding domain proteins (MBD) serves as an important mediator to couple DNA methylation to repressive chromatin configurations. The expression of the family members *Mecp2*, *Mbd1* and *Mbd2* increased concurrently to *Dnmt3a* during differentiation (Figure 5A), whereby increases in *Mbd1* and *Mbd2* surpassed the one of *Mecp2*. Interestingly, however, this led only to an increase of Mecp2, but not of Mbd1 and Mbd2, occupancy at the enhancer (Figure 5C, data not shown) and thus corroborated our previous finding that Mecp2 and other MBDs bound in a rather mutually exclusive manner across the *Avp* locus. This behavior is likely to reflect the presence of high-affinity MeCP2 binding sites at the *Avp* enhancer region as previously described [6], [23].

Histone deacetylases mediate transcriptional repression by PcG [24], Dnmts [25] and also Mecp2 [26]. Consistent with this view, Lif withdrawal halved Hdac1 and Hdac2 occupancy at the *Avp* enhancer and supported the hypothesis that PcG binding contributes to the recruitment of Hdacs at the *Avp* enhancer.

### Mecp2 binding depends on prior Suz12 occupancy

**Figure 6:**
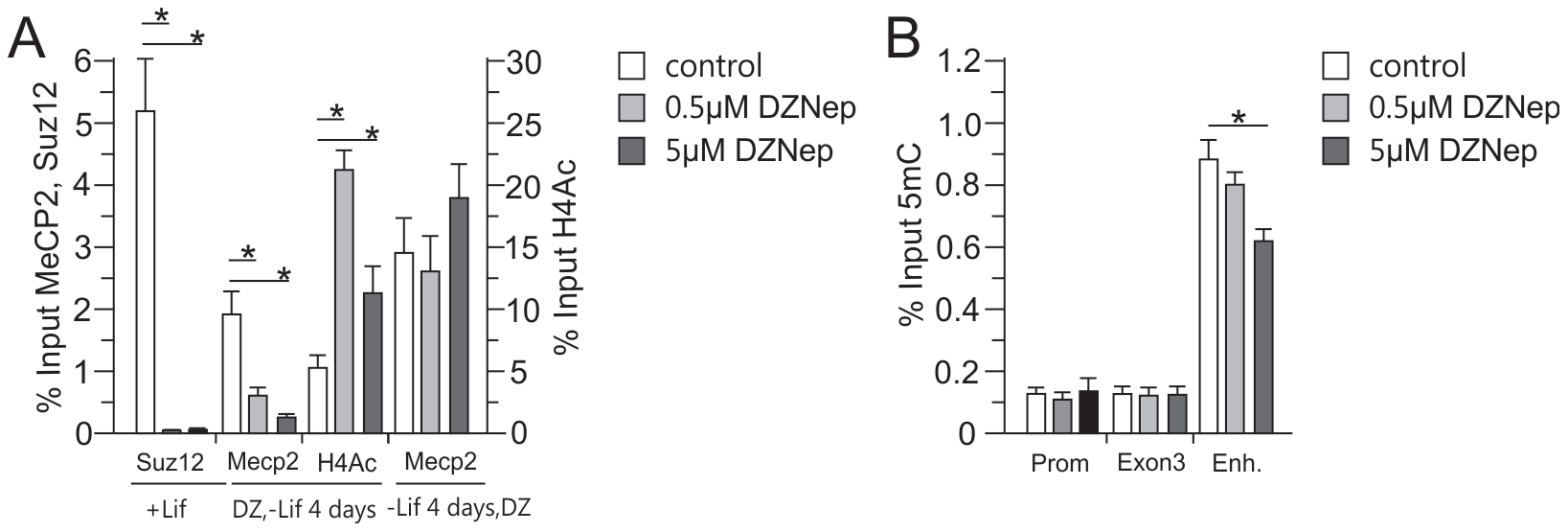
A, DZNep treatment inhibited Suz12 occupancy and Mecp2 recruitment at the *Avp* enhancer during hypothalamic-like differentiation of EB5 cells. Chromatin samples from cells treated with DZNep during differentiation were immunoprecipitated with antibodies against Suz12, Mecp2 or H4Ac and recovered DNA analyzed by qPCR for the presence of the *Avp* enhancer. Cells treated with DZNep (DZ) in the undifferentiated state for 24 hrs (+Lif) showed strongly reduced Suz12 binding. Following 4 days of differentiation (DZ,-Lif 4 days), Mecp2 binding was decreased while H4Ac was increased. In contrast, following 3 days of differentiation an additional 24 hours application of DZNep (-Lif 4days, DZ) did not affect Mecp2 occupancy. B, DZNep treatment led to reduced methylation at the *Avp* enhancer. MeDIP evidenced reduced 5hmC at the downstream enhancer in response to increasing doses of DZNep, while methylation levels at the promoter and gene body regions did not change. Bars represent standard deviations from the mean (sem), *, *P*<0.05; from 5 independent experiments performed.

Concurrent to the loss of Suz12 occupancy (Figure 3B), Mecp2 binding at the *Avp* enhancer increased during hypothalamic-like differentiation (Figure 5C). In order to understand the relationship between these two factors, we tested the effects of DZNep, a potent S-adenosylhomocysteine (AdHcy) hydrolase inhibitor that leads to the depletion of Suz12 and PRC2 protein complex formation [27]. In accord with this report, treatment of naïve EB5 cells with DZNep resulted in decreased Suz12occupancy at the *Avp* enhancer and a subsequent reduction in Mecp2 recruitment during hypothalamic-like differentiation. In contrast, DZNep treatment of differentiated cells elicited no effect on Mecp2 binding at the *Avp* enhancer, suggesting that depletion of Suz12 during the embryonic state is an important step prior to Mecp2 recruitment (Figure 6A). Compatible with this view, PcG protein complexes can directly interact with Dnmts and recruit them to their target genes [28], [29]. In support of this scenario, MeDIP analysis of DZNep treated EB5 cells showed reduced methylation of the ELS-responsive CpG residues at the *Avp* enhancer region when applied prior to differentiation (Figure 6B).

### Mecp2 binding associates with repressive chromatin marks at the *Avp* enhancer

Mecp2 had been originally identified as a protein that specifically binds to methylated DNA to confer gene silencing. More recent work suggested, however, an additional role as activator of gene transcription in hypothalamic tissues (*for review see* [30]).

**Figure 7:**
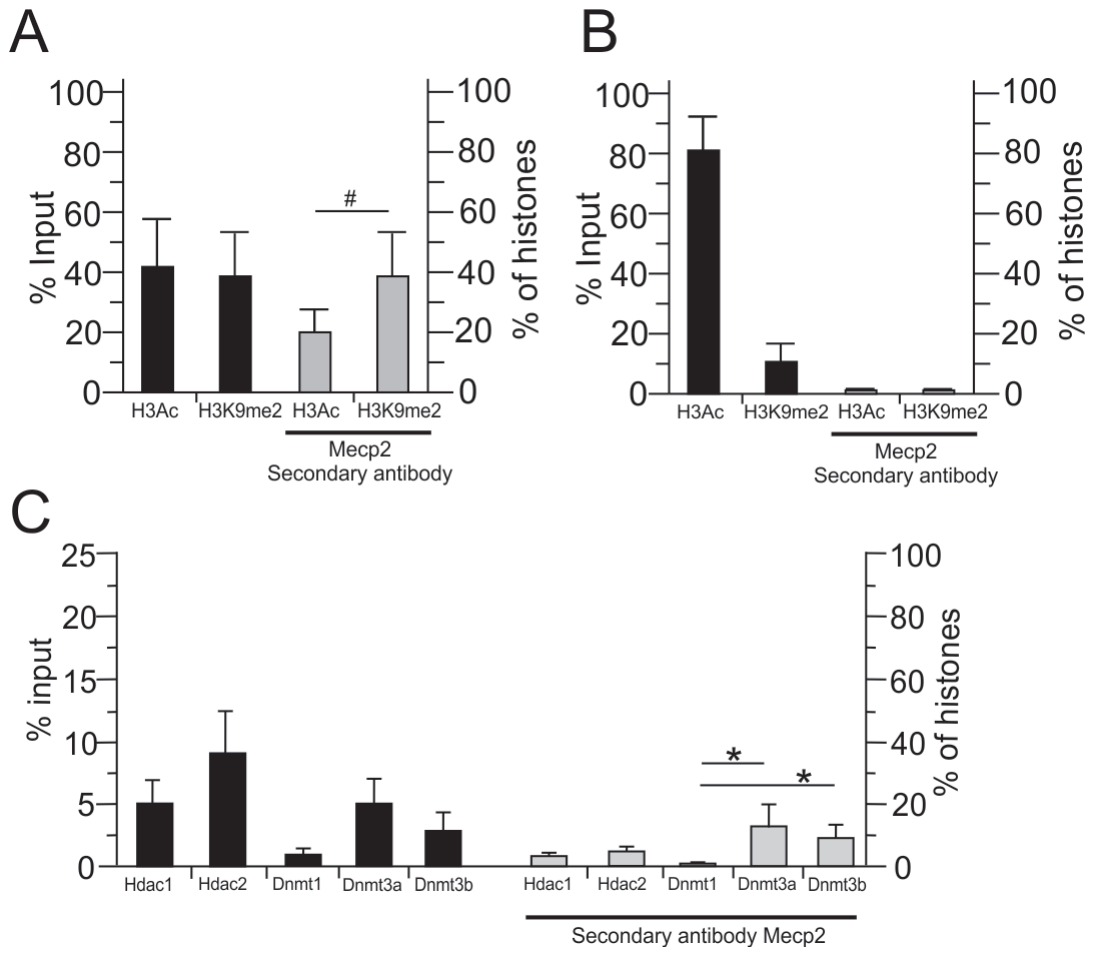
Mecp2 occupancy at the *Avp* enhancer coupled with repressive chromatin marks and repressor complexes. A, Chromatin samples from the PVN were subjected to seqChIP. The first ChIP was done with antibodies against H3Ac (panacetylated-histone H3), an active mark, and H3K9me2 (dimethyl-histone H3, Lys-9), a repressive mark. The second round ChIP was carried out with anti-Mecp2. Hereby, Mecp2 was preferentially, with a distinct trend toward significance, contained in the chromatin fraction associated with the transcriptionally inactive *Avp* enhancer. B, PVN tissues from Mecp2 null male (-/y) mice showed increased H3Ac and decreased H3K9me2 chromatin marks at the *Avp* enhancer compatible with a repressor function of Mecp2. However, due to the knockout of *Mecp2*, the anti-Mecp2 antibody did not enrich for either chromatin fraction attesting to its specificity. C, Sequential ChIP experiments showed that Mecp2 occupancy at the ELS-responsive enhancer associated with the chromatin fraction containing Hdacs and Dnmts. Chromatin samples from hypothalamus were immunoprecipitated in the first ChIP experiment with antibodies against Hdac1, Hdac2, Dnmt1, Dnmt3a and Dnmt3b. Following on two-thirds of the product of the primary ChIP were precipitated in the second ChIP with anti-C-terminal Mecp2; the remaining sample was used for the analysis of the primary ChIP. DNA recovered from both ChIP steps was analyzed by qPCR for the presence of the *Avp* enhancer. Bars represent standard deviations from the mean (sem), *, *P*<0.05; #, *P*<0.07 from 5 independent experiments performed.

In view of the finding from above that Mecp2 binding at the *Avp* enhancer followed polycomb silencing and that Avp expression increased at the same time, we asked whether Mecp2 acts to enhance *Avp* expression, or alternatively, to restrain expression subsequent to the dissociation of the repressor complex. Using sequential ChIP (seqChIP) we found Mecp2 preferentially in the chromatin fraction precipitated with H3K9me2, a mark of transcriptionally inactive chromatin, when compared to H3Ac (panacetyl-histone H3), an active mark, in both the PVN (Figure 7A) and SON (data not shown), demonstrating enrichment of Mecp2 in chromatin containing repressive histone marks. Mecp2 deficient mice (-/y) served as a control for the specificity of the Mecp2 antibody in seqChIP experiments (Figure 7B). Moreover, we detected about 2-fold higher amounts of the active mark H3Ac concomitant with about 3-fold lower amounts of the repressive mark H3K9me2 in Mecp2 deficient mice (-/y) consistent with the hypothesis that Mecp2 occupancy favors the formation of a repressive chromatin complex at the *Avp* enhancer (Figure 7B).

**Figure 8:**
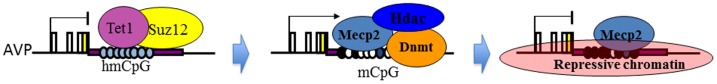
Model for the formation of early-life stress responsive DNA methylation. In the undifferentiated state, Tet1 and polycomb complexes (for the sake of simplicity symbolized by the histone methyltransferase Suz12) bind to the *Avp* enhancer and confer gene silencing. Hereby, Tet1 catalyzes the hydroxylation of methylated CpG residues (mCpG → 5hmC) in favor of further demethylation and maintained polycomb occupancy. Upon hypothalamic-like differentiation, polycomb and Tet1 proteins dissociate and lead to an increase in *Avp* enhancer methylation. Subsequently, recruitment of Mecp2 together with Hdacs and Dnmts enforces the formation of CpG methylation and repressive histone marks restraining *Avp* expression. Overall, *Avp* enhancer methylation does not serve as an ‘on-off switch’ but enables programming of *Avp* expression in response to early-life stress.

Compatible with these findings, Mecp2 associated with the chromatin fraction containing the de novo methyltransferases Dnmt3a and Dnmt3b, but not Dnmt1, and to less degree with Hdac 1 and 2 at the *Avp* enhancer in mouse hypothalamus (Figure 6C). Together, these results suggest that Mecp2 binding at the *Avp* enhancer following polycomb displacement promotes the recruitment of Dnmts and Hdacs (Figure 8) and thus opens up the possibility to relieve gene repression through ELS-dependent phosphorylation and dissociation of Mecp2.

## Discussion

Here we showed that PcG occupancy at the *Avp* locus silences early gene expression and precedes the emergence of ELS-responsive DNA methylation at the enhancer region. Dissociation of PcG complexes triggered DNA methylation and subsequent Mecp2 occupancy. Consistent with a role to restrain *Avp* expression, Mecp2 bound to chromatin enriched in Dnmts and Hdacs. Together, these findings extend the spectrum of action of PcG proteins to prime experience-dependent DNA methylation.

Given the tiny amounts of hypothalamic tissue available from embryonic mice precluding most of the molecular experiments reported here, we opted for a cellular model of hypothalamic-like differentiation to conduct a comprehensive series of functional studies. In parallel, correlations with mice hypothalamic development were sought for by appropriate gene expression and molecular studies wherever feasible.

Using an ESC-derived model of hypothalamic-like differentiation we discovered that *Avp* silencing in the undifferentiated state concurred with PcG occupancy at the gene body and ELS-responsive enhancer region. PcG proteins were originally identified as part of a stable, epigenetic cellular memory system that controls gene silencing via chromatin structure. In addition to their role as epigenetic gatekeepers, recent reports suggest that PcG proteins are also involved in controlling dynamics and plasticity of gene regulation, particularly during differentiation, by interacting with various components of the transcriptional machinery [19]. These findings raised the possibility that PcG protein complexes also contribute to experience-dependent programming of *Avp* expression. In the present study, we showed, however, that following hypothalamic-like differentiation of ESCs, PcG proteins dissociated from the *Avp* locus concomitant to de novo DNA methylation at the downstream enhancer.

Consistent with a functional link between PcG occupancy and de novo methylation [32], depletion of PcG proteins through DZNep treatment prior to hypothalamic-like differentiation reduced the appearance of ELS-responsive methylation at the *Avp* enhancer. PcG protein complexes associate with Tet proteins [31], Hdacs [24] and also Dnmts [28], [29], whereby the balance between Tet proteins (driving demethylation) and Dnmts (driving methylation) is critical to the maintenance or loss of PcG occupancy [31].

Although we evidenced PcG occupancy at the *Avp* gene body (high-CpG frequency island) and the downstream enhancer (intermediate-CpG frequency island), Dnmt3a occupancy and de novo methylation was targeted to the *Avp* enhancer consistent with the idea that de novo methylation of CpG islands occurs primarily at weak-CpG islands leading to gene silencing [32]. In further support of our present findings non-promoter methylation by Dnmt3a has been implicated as critical in antagonizing PcG repression and inducing active chromatin states of neurogenic genes [33].

Treatment with the Shh antagonist cyclopamine, giving rise to PVN/SON-like neurons, promoted *Avp* expression despite preserved enhancer methylation. Moreover, *Avp* expression and enhancer methylation increased both postnatally until adulthood. Together, these findings suggest that DNA methylation at the *Avp* enhancer does not fulfill a role as an ‘on-off’ switch of gene expression. Instead, *Avp* enhancer methylation can serve to fine-tune gene expression levels in response to environmental signals.

In support of this view, hypothalamic *Mecp2* expression increased during perinatal/postnatal development in the mouse and rat [34]; a period crucial for epigenetic programming of *Avp*. Upon binding to the *Avp* enhancer, Mecp2 associated with Hdacs and Dnmts, in particular Dnmt3a, the expression of which increased perinatally in the hypothalamus. As a result, Mecp2 occupancy at the *Avp* enhancer correlated with repressive chromatin marks supporting its role in repression [6].

Understanding the molecular basis by which cells form and maintain their memory represents an ongoing task. Epigenetic mechanisms such as DNA methylation can alter gene expression in response to the environment and thus the intrinsic properties of neurons in a long-term fashion, perhaps on the order of years [9], [35]. Here we provide insight in the mechanisms leading to ELS-responsive DNA methylation at the *Avp* enhancer in an in-vitro model of hypothalamic-like differentiation and assign an important role to PcG proteins. In this respect, our present work describes how mechanisms operating during early cellular development (i.e. PcG proteins) intersect with mechanisms mediating early-life experiences (i.e. Mecp2). Overall, we suggest that cell memories can evolve gradually over time by recruiting and building up on the mechanism available at a given age.

## Supporting Information

File S1 **Figures S1 and S2 and Tables S1–S3.**
(DOC)
