# Supplementary material for: Polycomb Binding Precedes Early-Life Stress Responsive DNA Methylation at the Avp Enhancer
Source: PLoS One. 2014 Mar 5;9(3):e90277. doi: 10.1371/journal.pone.0090277 (PMC3943912; doi:10.1371/journal.pone.0090277)
Supplement: File S1 — Figures S1 and S2 and Tables S1–S3. (DOC) [file pone.0090277.s001.doc]

Polycomb binding precedes early-life stress responsive DNA methylation at the *Avp* enhancer

**Chris Murgatroyd^1,2^* and Dietmar Spengler^1^**

^1^ Max-Planck Institute of Psychiatry, Molecular Neuroendocrinology, Kraepelinstr. 2-10,

80804 Munich, Germany

^2^ Manchester Metropolitan University, Manchester, M1 5GD, UK

**Contents**

The file contains Supplementary Figures S1 to S3, and Supplementary Tables 1 to 3.

**Supplementary Figures**

Figure S1 | DNA methylation at the *Nanog* promoter and repeat elements upon differentiation 2

Figure S2 | Suz12 occupancy at *Wnt1* and *RNAPII* promoters upon differentiation 2

**Supplementary Tables**

Table S1 | RT-PCR primer sequences. 3

Table S2 | Bisulphite primer sequences. 4

Table S3 | ChIP primer sequences. 4


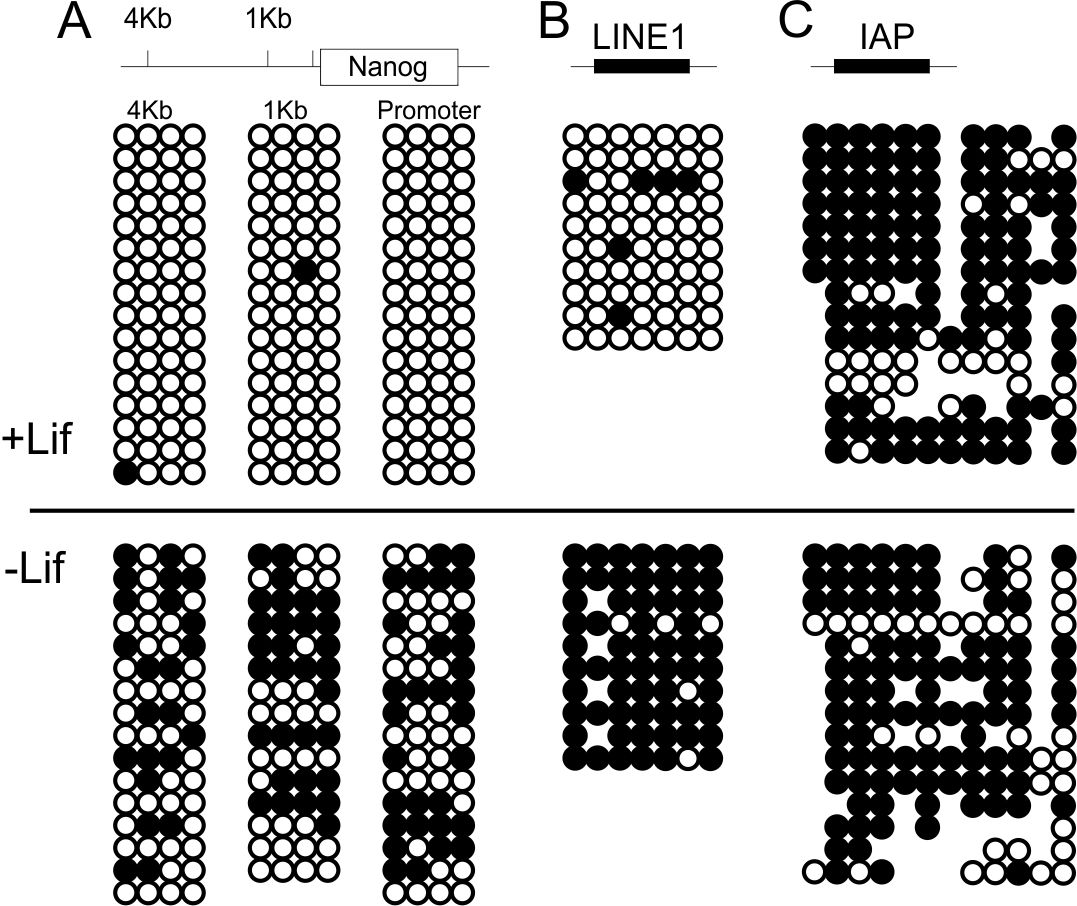


Figure S1 | DNA methylation at the *Nanog* promoter and repeat elements upon hypothalamic-like differentiation of EB5 cells. A, Following Lif withdrawal for 4 days (-Lif), CpG methylation increased across the *Nanog* promoter corresponding with transcriptional silencing of the gene (Figure 1A, main text). B, Similarly, the *LINE1* repeat element gained in DNA methylation following 4 days of neuronal differentiation. C, In contrast, DNA methylation at the IAP repeat element did not change in response to Lif-withdrawal.


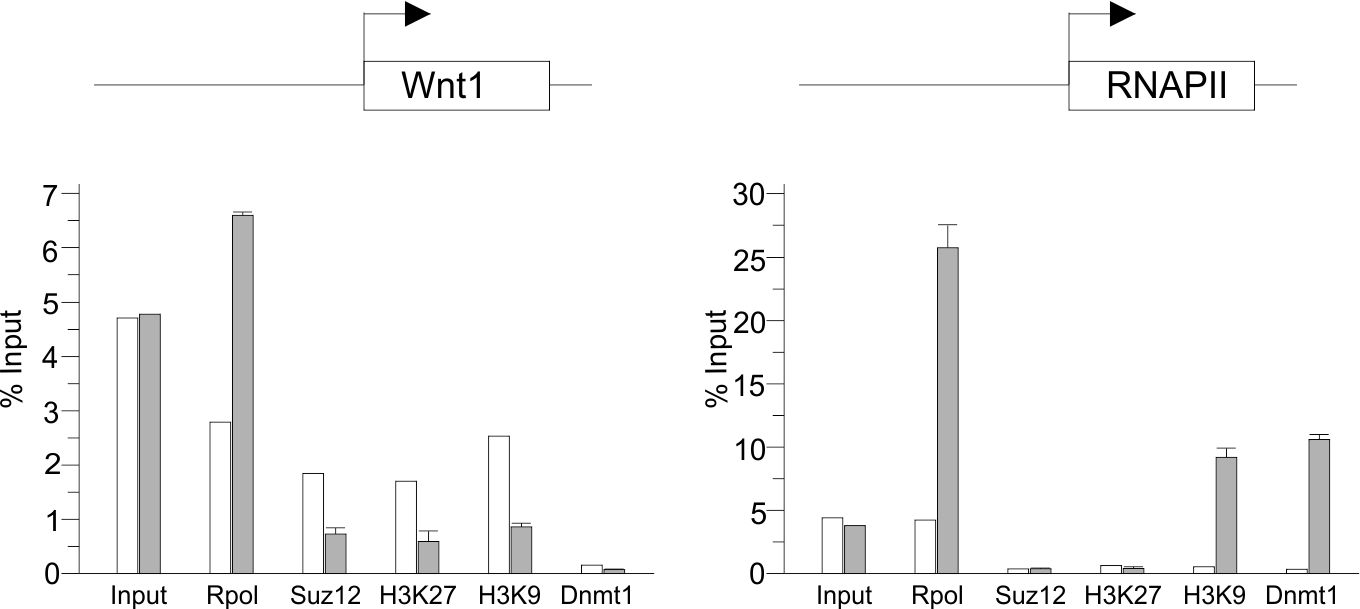


Figure S2 | Suz12 occupancy at *Wnt1* and *RNAPII* promoters upon hypothalamic-like differentiation. Suz12 binding at the *Wnt1* (wingless) promoter, a known target of PcG complexes, decreased following 4 days of Lif-withdrawal. In contrast, Suz12 occupancy at the *RNAPII* (RNA polymerase II) promoter, a house-keeping gene, remained unaffected.

Supplementary Table 1. RT-PCR Primer sequences

| **Gene** | **sequences** |
| --- | --- |
| *Tet1* | F, gagcctgttcctcgatgtgg; R, caaacccacctgaggctgtt |
| *Tet2* | F, tgttgttgtcagggtgagaatc; R, tcttgcttctggcaaacttaca |
| *Tet3* | F, ccggattgagaaggtcatctac; R, aagataacaatcacggcgttct |
| *Vax1* | F, ttctgcttagtccgccgattc; R, gcccaagaggactcgcacg |
| *En2* | F, tctcaagccagcgccactc; R, gaaggctgtgcgaggccgc |
| *Irx* | F, gtgctttcatcagtgtacg; R, gaacgcggcggccgtggc |
| *Otx2* | F, ctccattctgctgctgctgc; R, ggaagctctgtttgccaagac |
| *Brn2* | F, actctcaccacctccttctc; R, gactcatcctcgggcagc |
| *Foxg1* | F, gccgccctcagcccagcttc; R, tgtccagcatcacccaggcatca |
| *Gli1* | F, ggaagtcctattcacgccttga; R, caaccttcttgctcacacatgtaag |
| *Shh* | F, gaagatcacaagaaactccgaacg; R, tggattcatagtagacccagtcgaa |
| *Otp* | F, gccgacctcctggacgccaggc; R, tgtctgggtctttggcgctcac |
| *Mecp2* | F, acagcggcgctccattatc; R, cccagttaccgtgaagtcaaaa |
| *MBD1* | F, agacggcagatgctccagagg; R, cccaggctgaaaatctccgtg |
| *MBD2* | F, gatgaagacattaggaaacagg; R, gtccatgtcaatgtctacttcc |
| *Patch1* | F, ctctggagcagatttccaagg; R, tgccgcagttcttttgaatg |
| *NPY* | F, tgtttgggcattctggctgagg; R, ttctgggggcgttttctgtgct |
| *Sim1* | F, gaggcaggcaggtactt; R, ctgaccacactatcttcat |
| *Nkx2.1* | F, ccaggacaccatgcggaaca; R, ggccatgttcttgctcacgt |
| *NeuroD1* | F, aagccatgaatgcagaggaggact; R, agctgcaggcagccggcgacc |
| *Arnt2* | F, gcaccggcaacaaatcg; R, agatgcttcagttcctgctcagt |
| *Six3* | F, ggtttaagaaccggcgacag; R, taccgagaggatcgaagtgc |
| *Rax* | F, ttcgagaagtcccactaccc; R, ttcatggacgacacttccag |
| *Nanog* | F, agggtctgctactgagatgctctg; R, caaccactggtttttctgccaccg |
| *Wnt1* | F, atgaaccttcacaacaacgag; R, ggttgctgcctcggttg |
| *Avp* | F, gctctccgcttgtttcctga; R, tgggcagttctggaagtagca |
| *Crh* | F, ggagccgcccatctctct; R, tgcccgggccatttc |
| *Pax6* | F, taacggagaagactcggatgaagc; R, cgggcaaacacatctggataatgg |
| *Oxt* | F, ggagacacttgcgcatatcca; R, ctgctacatccagaactgccc |
| *Sox2* | F, ggcagctacagcatgatgcaggagc; R, ctggtcatggagttgtactgcagg |
| *Dnmt1* | F, ggaaggctacctggctaaagtcaag; R, actgaaagggtgtcactgtccgac |
| *Dnmt3a* | F, tggagaatggctgctgtgtgac; R, cactcatcccgtttccgtttg |
| *Dnmt3b* | F, agtgaccagtcctcagacacgaag; R, atcagagccattcccatcatctac |
| *Nestin* | F, ctgcaggccactgaaaagtt ; R, gacccctgcttctcctgctc |
| *Atp5j* | F, tattggcccagagtatcagca ; R, ggggtttgtcgatgacttcaaat |
| *Gapdh* | F, ccatcaccatcttccaggagcgag; R, gatggcatggactgtggtcatgag |
| *B-actin* | F, tccatcatgaagtgtgacgt; R, gagcaatgatcttgatcttcat |
| *Hprt* | F, acctctcgaagtgttggatacagg; R, cttgcgctcatcttaggctttg |

Abbreviations: F, forward; R, reverse.

**Supplementary Table 2.** Bisulphite PCR Primer sequences

| **Gene** | **Sequence** |
| --- | --- |
| *Avp* promoter | F, cactgacacgcccacgtgtgtc; R, ggagagcgtagtgttgagcatc |
| *Avp* CpG island | F, gagctcttccttcagagagctgc; R, ggcgatggctcagtagacccg |
| *Avp* enhancer | F, gaaggtacagagatgttac; R, ggaccaaattcaggccag |
| *Nanog* 4KB | F, ggtagtttgttgggttttgtatttt; R, aactcttatctccccattcctaaac |
| *Nanog* 1Kb | F, ggaagattaggagtttgggattagt; R,atctaccaccatacccaatttaaaa |
| *Nanog* promoter | F, tttgtaggtattaattgtgaat; R, aaaaaaacaaaacaccaaccaaat |
| *IAP* | F, ttgatagttgtgttttaagtggtaaataaa; R, aaaacaccacaaaccaaaatcttctac |
| *LINE* | F, gttagagaatttgatagtaaatggaatagg; R, ccaaaacaaaacctttctcaaacactatat; nested R, tcaaacactatattactttaacaattccca |

**Supplementary Table 3.** ChIP Primer sequences

| **Gene** | **Sequence** |
| --- | --- |
| *Avp* promoter | F, cactgacacgcccacgtgtgtc; R, ggagagcgtagtgttgagcatc |
| *Avp* CpG island | F, gagctcttccttcagagagctgc; R, ggcgatggctcagtagacccg |
| *Avp* enhancer | F, gaaggtacagagatgttac; R, ggaccaaattcaggccag |
| *RNAPII* | F, gacgggttctgagcacttag; R, caacatcagcatcactgacc |
| *Wnt1* | F, gacggttagcctgtcagctc; R, ctgacgactgtggttgctgt |
